# *In vitro*/*vivo* Mechanism of Action of MP1102 With Low/Nonresistance Against *Streptococcus suis Type 2 Strain CVCC 3928*

**DOI:** 10.3389/fcimb.2019.00048

**Published:** 2019-02-26

**Authors:** Fei Zhao, Na Yang, Xiumin Wang, Ruoyu Mao, Ya Hao, Zhanzhan Li, Xiao Wang, Da Teng, Huan Fan, Jianhua Wang

**Affiliations:** ^1^Gene Engineering Laboratory, Feed Research Institute, Chinese Academy of Agricultural Sciences, Beijing, China; ^2^Key Laboratory of Feed Biotechnology, Ministry of Agriculture and Rural Affairs, Beijing, China; ^3^Tianjin Animal Science and Veterinary Research Institute, Tianjin, China

**Keywords:** *Streptococcus suis*, antimicrobial peptides, MP1102, membrane damage, DNA interference, *in vivo* efficacy

## Abstract

Streptococcosis is recognized as a leading infectious disease in the swine industry. *Streptococcus suis* serotype 2 is regarded as the most virulent species, which threatens human and pig health and causes serious economic losses. In this study, multiple *in vitro* and *in vivo* effects of MP1102 on multidrug resistant *S. suis* was studied for the first time. MP1102 exhibited significant antibacterial activity against *S. suis* (minimum inhibitory concentration, MIC = 0.028–0.228 μM), rapid bacteriocidal action, a longer postantibiotic effect than ceftriaxone, and a synergistic or additive effect with lincomycin, penicillin, and ceftriaxone (FICI = 0.29–0.96). No resistant mutants appeared after 30 serial passages of *S. suis* in the presence of MP1102. Flow cytometric analysis and electron microscopy observations showed that MP1102 destroyed *S. suis* cell membrane integrity and affected *S. suis* cell ultrastructure and membrane morphology. Specifically, a significantly wrinkled surface, intracellular content leakage, and cell lysis were noted, establishing a cyto-basis of nonresistance to this pathogen. DNA gel retardation and circular dichroism analysis indicated that MP1102 interacted with DNA by binding to DNA and changing the DNA conformation, even leading to the disappearance of the helical structure. This result further supported the mechanistic basis of nonresistance via interaction with an intracellular target, which could serve as a means of secondary injury after MP1102 is transported across the membrane. Upon treatment with 2.5–5.0 mg/kg MP1102, the survival of mice challenged with *S. suis* was 83.3–100%. MP1102 decreased bacterial translocation in liver, lung, spleen, and blood; inhibited the release of interleukin-1β and tumor necrosis factor-α; and relieved the lung, liver, and spleen from acute injury induced by *S. suis*. These results suggest that MP1102 is a potent novel antibacterial agent for the treatment of porcine streptococcal disease.

## Introduction

*Streptococcus suis* is a major porcine pathogen related to a series of serious zoonosis, including pneumonia, sepsis, meningitis, arthritis, and endocarditis, and can be transmitted to humans (Lun et al., 2007). *S. suis* serotype 2 is the most prevalent serotype and is the most closely associated with diseases in pigs and humans (Lun et al., 2007; Groves et al., 2015). In fact, its epidemic is very serious as a long-standing health problem worldwide. In the last decade, the number of cases reported was tripled (Zanen and Engel, 1975; Spiss et al., 1999; Hughes et al., 2009; Feng et al., 2010; Michaud et al., 2016). In particular, two large-scale outbreaks of human infections with *S. suis* disease in China in 1998 and 2005 resulted in a significant health crisis and loss (Yu et al., 2006; Feng et al., 2010). Intensive concerns worldwide, including the threat to human and animal health and significant economic loss, represent an area of wide focus to enhance the identification of effective prevention and control approaches against *S. suis* infections (Dejace et al., 2017).

At present, prevention and control measures of *S. suis* infection mainly include antibiotics and vaccines (Feng et al., 2014). Antibiotics have always played a leading role in therapy of diseases caused by *S. suis*. However, antibiotic resistance becomes increasingly serious due to antibiotic overuse and the single target nature of this class of drugs. Approximately 85% of *S. suis* strains are resistant to commonly used antibiotics, including macrolides, lincosamides, sulfonamides, and tetracyclines (Callens et al., 2013; Varela et al., 2013). Various types of vaccines have been developed, but their efficacy varies considerably. Immunization of pigs is hindered by the lack of an autogenous vaccine protecting against more than one serotype owing to the complexity of *S. suis* with multiple serotypes. Several proteins and factors from *S. suis* were discovered as vaccine candidates in succession, including muramidase-released protein, extracellular factor, suilysin, and immunoglobulin M-degrading enzyme. In addition, the benefits and limitations of these agents are also described (Wisselink et al., 2001; Kock et al., 2009; Seele et al., 2015; Segura, 2015). Currently, no effective commercial vaccine is available.

Therefore, effective antimicrobial agents against *S. suis* are urgently needed. Antimicrobial peptides (AMPs) have been receiving increased attention. A fungal defensin plectasin isolated from *Pseudoplectania nigrella* is especially active against Gram-positive bacteria, especially *Staphylococcus* and *Streptococcus* (Mygind et al., 2005). MP1102, a plectasin-derived peptide, displays more potent antibacterial activity, low hemolytic activity (0.05% hemolysis at a concentration of 128 μg/ml) and strong *in vivo* bactericidal activity toward *S. aureus* (Zhang et al., 2015), indicating that it is a potential candidate mainline drug against bacterial infection. However, no studies have focused on the activity of MP1102 against *S. suis*.

From the antimicrobial susceptibility results of the disk diffusion method (Table 1) (Clinical Laboratory Standard Institute, 2014), *S. suis* CVCC 3928 showed resistance to neomycin, sulfisoxazole, and polymyxin B and sensitivity to cefazolin, streptomycin, tetracycline, erythromycin, azithromycin, vancomycin, chloramphenicol, norfloxacin, and penicillin. In this work, *in vitro* dual mechanisms of MP1102 against *S. suis* CVCC 3928 with significantly high antibacterial activity and low/nonresistance were elucidated for the first time; additionally, *in vivo* effects of MP1102 treatment were evaluated through a mouse peritonitis model induced by *S. suis*.

**Table 1:** The susceptibility analysis of *S. suis* CVCC 3928 against antimicrobial.

**Antimicrobial**	**Disc content**	**S (mm)**	**I (mm)**	**R (mm)**	**Measured value (mm)**	**Susceptibility evaluation**
Cefazolin	30 μg	≥18	15–17	≤ 14	37.5	S
Gentamycin	10 μg	≥15	13–14	≤ 12	14.5	I
Kanamycin	30 μg	≥18	14–17	≤ 13	13.5	I
Streptomycin	10 μg	≥15	12–14	≤ 11	16.5	S
Neomycin	30 μg	≥17	13–16	≤ 12	11.5	R
Tetracycline	30 μg	≥23	19–22	≤ 18	26	S
Erythromycin	15 μg	≥21	16–20	≤ 15	26.5	S
Azithromycin	15 μg	≥18	14–17	≤ 13	24.5	S
Vancomycin	30 μg	≥17			22.5	S
Polymyxin B	300 IU	≥12	9–11	≤ 8	0	R
Chloramphenicol	30 μg	≥18	13–17	≤ 12	28	S
Bacitracin	0.04 U	≥13	9–12	≤ 8	11.5	I
Sulfisoxazole	300 μg	≥19	16–18	≤ 15	14.5	R
Norfloxacin	10 μg	≥17	13–16	≤ 12	20.5	S
Penicillin	10 U	≥28	20–27	≤ 19	36.5	S

*R, resistant; I, intermediate; S, sensitive*.

## Materials and Methods

### Materials

MP1102 with the purity of 96.4% was prepared based on our previous protocols (Zhang et al., 2015) and stored at −20°C before subsequent antibacterial assessments. Ceftriaxone and propidium iodide (PI) were purchased from Dalian Meilun Biological Technology Co., Ltd. and Sigma-Aldrich Shanghai Trading Co. LLC (Shanghai, China), respectively. Other reagents were of analytical grade. *S. suis* strains (CVCC 3928, 3309, and 606) isolated from piglets and *Streptococcus pneumoniae* CVCC 2350 were obtained from the China Veterinary Culture Collection (CVCC). *S. pneumoniae* CGMCC 1.8722 was obtained from China General Microbiological Culture Collection Center (CGMCC). Mice were purchased from Beijing Vital River Laboratory Animal Technology Co., Ltd.

### Determination of Minimum Inhibitory Concentration (MIC)

The MICs of MP1102 were measured by the broth microdilution method (Yang et al., 2016). Test strains were cultured to mid-log phase at 37°C in Mueller-Hinton Broth medium (MHB, Beijing Aoboxing Bio-Tech Co. Ltd.) and diluted with MHB medium to 1 × 10^5^ CFU/ml. A 10-μl of serial two-fold dilutions of MP1102 or ceftriaxone and 90 μl cell suspension were added to each well of 96-well microplates and then incubated at 37°C for 18-24 h. The MIC was defined as the lowest concentration that prevented visible growth of bacteria. All assays were performed in triplicate.

### Bactericidal Kinetics Measurement

Bacterial cells were grown to exponential-phase at 37°C in MHB medium and incubated with different concentrations of MP1102 (1 ×, 2 ×, and 4 × MIC, respectively). Ceftriaxone and PBS were used as the positive and blank controls, respectively. The samples were incubated at 250 rpm and 37°C. The 100-μl samples were taken from each flask at different time intervals (0–10 h) and plated to count the colonies after gradient dilution with PBS (Xi et al., 2013).

### Membrane Permeabilization Analysis by Flow Cytometer

*S. suis* strain CVCC 3928 cells were cultured to mid-log phase at 37°C in MHB medium, washed with 0.01 M PBS buffer thrice, resuspended in the PBS buffer to 1 × 10^7^ CFU/ml and incubated with 1 ×, 2 ×, and 4 × MIC MP1102 at 37°C for 0.5 and 2 h. The samples were detected by a FACS Calibur Flow Cytometer (BD, USA) after fixation with PI, and CellQuest Pro software (BD, USA) was used for data analysis (Zong et al., 2016).

### Resistance to MP1102

Resistance to MP1102 and antibiotics (ceftriaxone, penicillin, and lincomycin) was assessed by the MIC assays. A mid-log phase culture of *S. suis* (1 × 10^5^ CFU/ml) (90 μl/well) was added into 96-well plates. Solutions of MP1102 or antibiotics (10 μl) were added into plates at concentrations of 8 ×, 4 ×, 2 ×, 1 ×, 0.5 ×, 0.25 ×, 0.125 ×, and 0.0625 × MIC. After 18 h incubation at 37°C under continuous shaking at each passage, cells from the second highest concentration showing visible growth were used to inoculate the subsequent culture. The serial passaging was repeated for 30 d (Mah et al., 2003; Li et al., 2017).

### The Postantibiotic Effect (PAE) of MP1102 Against *S. suis*

After treatment with MP1102 or ceftriaxone sodium (1 ×, 2 ×, and 4 × MIC) for 2 h, *S. suis* cells (10^7^ CFU/ml) were diluted 1,000 times by medium, transferred to new flasks and incubated at 37°C and 250 rpm. The samples were taken from flasks for counting every hour until bacterial cultures become turbid. Untreated bacteria were used as controls. The PAE was calculated using the equation: PAE = T-C, where T is time (h) required for the CFU in the test culture to increase by 10 times above the count immediately after dilution and C is the corresponding time (h) for the control (Li et al., 2017).

### Synergism Test

The checkerboard microtiter assay was applied for determining the interaction of combinations of MP1102 with ceftriaxone, penicillin, lincomycin, kanamycin, and gentamicin, separately (Zong et al., 2016). MP1102 and antibiotics were dispensed into 96-well cell culture plates at final concentrations ranging from 1/8 to 8 × MIC in each well. The MIC values were determined as the MIC assay described above. The effects of combination were evaluated by calculating the fractional inhibitory concentration index (FICI) of each combination. The synergistic experiments were performed in triplicate. The FICI represents the sum of the FICs of each drug tested, where the FIC is defined as the MIC of each drug when used in combination divided by the MIC of the drug when used alone. FICI = (MICdrug A in combination/MICdrug A alone) + (MICdrug B in combination/MICdrug B alone).

### Transmission Electron Microscope (TEM) Observations

The mid-logarithmic *S. suis* CVCC 3928 cells (1 × 10^8^ CFU/ml) and 4 × MIC MP1102 were incubated at 37°C for 2 h. The cells were fixed in 2.5% (w/v) glutaraldehyde; postfixed in 1% osmium tetroxide (OsO_4_); dehydrated with an ethanol series of 50, 70, 85, 95, and 100% (15 min/time); and transferred to epoxy resins (Wang et al., 2017). The cells were stained with 1% uranium acetate and observed with a TEM (JEM1400, Japan).

### Scanning Electron Microscopy (SEM) Observations

The mid-logarithmic *S. suis* CVCC 3928 cells (1 × 10^8^ CFU/ml) and 4 × MIC MP1102 were incubated at 37°C for 2 h. The cells were fixed with 1% OsO_4_, dehydrated by ethanol series (50, 70, 85, 95, and 100%) and dried by CO_2_ according to the previous study (Zong et al., 2016). After platinum coating, the samples were observed with a QUANTA200 SEM (FEI, Philips, Netherlands).

### Interaction of MP1102 With the Genomic DNA From *S. suis*

The gel retardation assay was performed to observe the binding of MP1102 to *S. suis* genomic DNA. Genomic DNA was extracted from *S. suis* strain CVCC 3928 using a bacterial DNA kit (TIANGEN Biotech Co., Ltd., Beijing). MP1102 was dissolved and serially diluted with DNA-binding buffer. The samples containing MP1102 and genomic DNA (0.5 μg) in 20 μl DNA binding buffer at peptide/DNA ratios ranging from 0.5 to 10.0 (w/w) were mixed at 37°C for 10 min. Genomic DNA was photographed using a Geliance 200 imaging system (PerkinElmer, USA) after electrophoresis (Hao et al., 2017).

Circular dichroism (CD) spectra were performed to examine whether MP1102-DNA binding affects the secondary structure of the genomic DNA from *S. suis* CVCC 3928. After the incubation of MP1102 and genomic DNA at mass ratios of 0, 0.5, and 10.0 for 10 min at room temperature, the mixtures were loaded into a cuvette of 1.0-mm path length and scanned from 220 to 300 nm at 25°C using a Pistar π-180 CD spectrometer (Applied Photophysics Ltd., UK) (Zong et al., 2016).

### Mouse *in vivo* Experiments

The *S. suis* infection protocol was performed as previously described (Jiao et al., 2017). The Institute for Cancer Research (ICR) female mice (6-week-old) (ten mice/group) were intraperitoneally injected with 1.0 ml of *S. suis* CVCC 3928 (7.5 × 10^8^ CFU/ml) and given two doses of MP1102 (2.5 and 5.0 mg/kg of body weight) or ceftriaxone (7.5 and 15.0 mg/kg of body weight) after 2 h postinfection. Mice injected with only *S. suis* CVCC 3928 or PBS served as negative or blank controls, respectively. Survival rates of mice were monitored daily for 7 d.

To assay bacterial loads in organs, the lungs, livers, and spleens were removed from the mice sacrificed at 24 h posttreatment and homogenized in sterile PBS for *S. suis* CVCC 3928 colony counting. Each group was repeated with five mice.

Serum was separated from mice 4 h posttreatment. The levels of cytokines (IL-1β, IL-10, and TNF-α) in serum were detected using an enzyme-linked immunosorbent assay (ELISA) kit from Jiaxuan Biotech Co., Ltd. (Beijing) (Wang et al., 2017). Each group was repeated with nine mice that were randomly subdivided into three groups for cytokines assay.

The mice were sacrificed 1 and 7 d posttreatment, and the lungs, livers and spleens were removed and fixed in 4% paraformaldehyde for 1 d. The organs were infiltrated with xylene and embedded in paraffin. Then, samples were sectioned and stained with hematoxylin eosin (HE). The tissue samples were observed using a light microscope (BX51, Olympus Optical Company, Japan). Subsequently, the degree of inflammation of tissues was graded in Beijing Jialanhai Biotech Co., Ltd. (Knodell et al., 1981; Rotta et al., 1999).

### Significance Analysis

The data were conducted using ANOVA with software SAS 9.2 (SAS Institute Inc., USA). The *P* < 0.05, *P* < 0.01, and *P* < 0.001 were defined as statistically significant, highly significant, and very highly significant, respectively.

### Ethics Statement

All animal procedures used in this study were approved by the Animal Care and Use Committee of Feed Research Institute, Chinese Academy of Agricultural Sciences (Permit Number: 20150309) and were performed in accordance with ARRIVE (Animals in Research: Reporting *in vivo* Experiments) guidelines (Kilkenny et al., 2010).

## Results

### MP1102 Exhibits Potent Antibacterial Activity Against *S. suis*

The antibacterial activity of MP1102 and ceftriaxone against the *Streptococcus* was listed in Table 2. The MICs of MP1102 against the test *S. suis* strains were from 0.028 to 0.228 μM, and those of ceftriaxone were 0.048 and 0.201 μM. For *S. pneumoniae* CGMCC 1.8722 and CVCC 2350, the MICs of MP1102 were 0.228 μM, however, ceftriaxone did not display obvious antibacterial activity against the test *S. pneumoniae* strains (MIC > 25.723 μM).

**Table 2:** Minimum inhibitory concentrations (MICs) assay.

**Strains**	**MP1102 (μM)**	**Ceftriaxone sodium (μM)**
*Streptococcus suis* CVCC 3928	0.028	0.048
*S. suis* CVCC 3309	0.028	0.096
*S. suis* CVCC 606	0.228	0.201
*Streptococcus pneumoniae* CGMCC 1.8722	0.228	>25.723
*S. pneumoniae* CVCC 2350	0.228	>25.723

### MP1102 Effectively Kills *S. suis*

**Figure 1:**
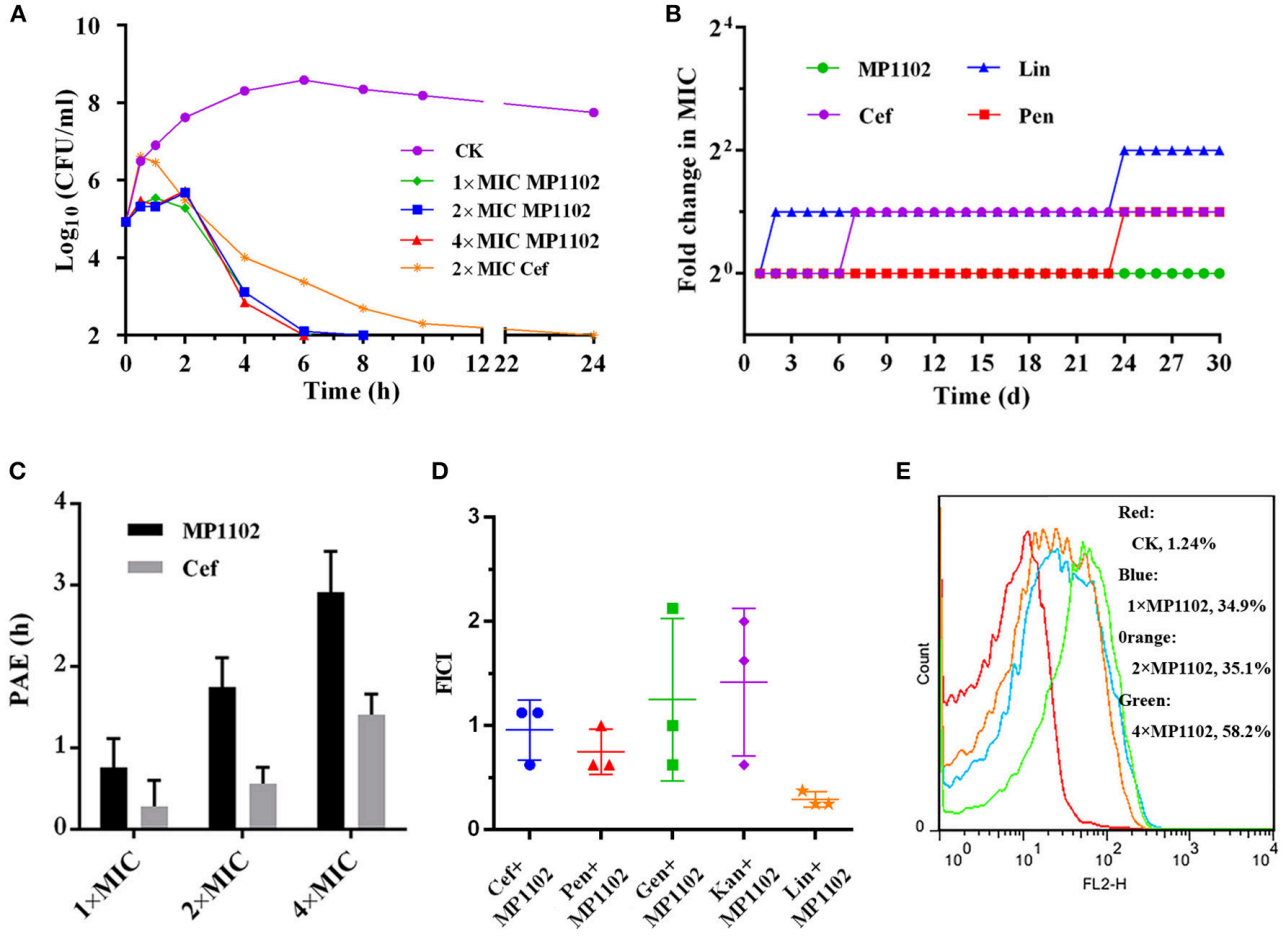
Bactericidal kinetics and flow cytometric analysis of MP1102. **(A)** Bactericidal kinetics assay of *S. suis* CVCC 3928 treated with MP1102. **(B)** Resistance of MP1102 and antibiotics; **(C)** PAE of MP1102 or ceftriaxone (Cef) (1 ×, 2 ×, and 4 × MIC); **(D)** Synergism test of MP1102 and antibiotics (Cef, ceftriaxone; Pen, penicillin; Lin, lincomycin; Kan, kanamycin; Gen, gentamicin); **(E)** Flow cytometric analysis of membrane permeabilization. After treatment with 1 ×, 2 ×, and 4 × MIC MP1102 for 2 h, *S. suis* CVCC 3928 cells were stained with PI and analyzed by flow cytometry.

As shown in Figure 1A, the *S. suis* cells counts (log_10_ CFU/ml) in the control group reached to 8.59 at 6 h and remained at a steady level from 8 to 24 h. For the MP1102 treatment groups, the cell counts decreased to complete sterilization in 6–8 h in a dose-independent manner, and no colonies were regrown even at 1 × MIC MP1102. However, the bacterial counts in the 2 × MIC ceftriaxone group showed slow reduction to complete sterilization until 24 h.

### Delayed Drug Resistance

Long-term and repeated exposures of microorganisms to subinhibitory doses of antibiotics promote acquisition of drug resistance (Hodges et al., 1992). To investigate whether MP1102 could adequately prevent drug resistance development, *S. suis* CVCC 3928 exposed to sub-MIC peptides were passaged daily and used for determination of MIC values for up to 30 passages. Various classes of clinically used antibiotics, including ceftriaxone, lincomycin, and penicillin, were used as controls in this study. As shown in Figure 1B, development of resistance was not observed in *S. suis* during continuous serial passaging in the presence of sub-MIC of MP1102 over 30 d. In contrast, *S. suis* rapidly developed resistance to lincomycin within 2 d of exposure. Additionally, resistance of ceftriaxone and penicillin increased by 2-fold on the second and 24th d.

### PAE of MP1102 in *S. suis*

The results of a dose-independent PAE of MP1102 or ceftriaxone against *S. suis* CVCC 3928 are shown in Figure 1C. The PAE values of MP1102 to *S. suis* were 0.76, 1.75, and 2.92 h at 1 ×, 2 ×, and 4 × MIC, respectively. The PAE values of ceftriaxone to *S. suis* were 0.28, 0.56, and 1.41 h at 1 ×, 2 ×, and 4 × MIC, respectively. The results indicated a dose-dependent PAE with MP1102 and ceftriaxone against *S. suis*, and the PAEs of ceftriaxone were reduced compared with those of MP1102.

### Synergism Assays

The efficacy of interactions between MP1102 and traditional antibiotics against *S. suis* CVCC 3928 is shown in Figure 1D. Combination between MP1102 and lincomycin showed a synergistic effect with FICI of 0.29. The FICI between MP1102 and ceftriaxone (FICI = 0.96) and penicillin (FICI = 0.75) showed an additive effect. In addition, kanamycin and gentamicin showed no effect with FICI indexes of 1.25 and 1.42. Antagonism was not detected.

### MP1102 Destroyed *S. suis* Cell Membrane Integrity

PI can intercalate into nucleic acids after penetrating the damaged cell membrane. Therefore, PI was used as an indicator to assay the effects of MP1102 on *S. suis* cell plasma membrane by flow cytometry (Xi et al., 2014). Figure 1E shows that the percentages of PI-permeable *S. suis* cells after treatment with 1 ×, 2 ×, and 4 × MIC MP1102 for 2 h were 34.9, 35.1, and 58.2%, respectively. However, in the absence of MP1102, the percentage of PI influx into the *S. suis* cells was 1.24%, suggesting that *S. suis* cell membranes were intact. These results indicated that MP1102 disrupted cell membrane with penetrating action in a concentration-dependent manner.

### MP1102 Affected *S. suis* Cell Ultrastructure and Membrane Morphology

**Figure 2:**
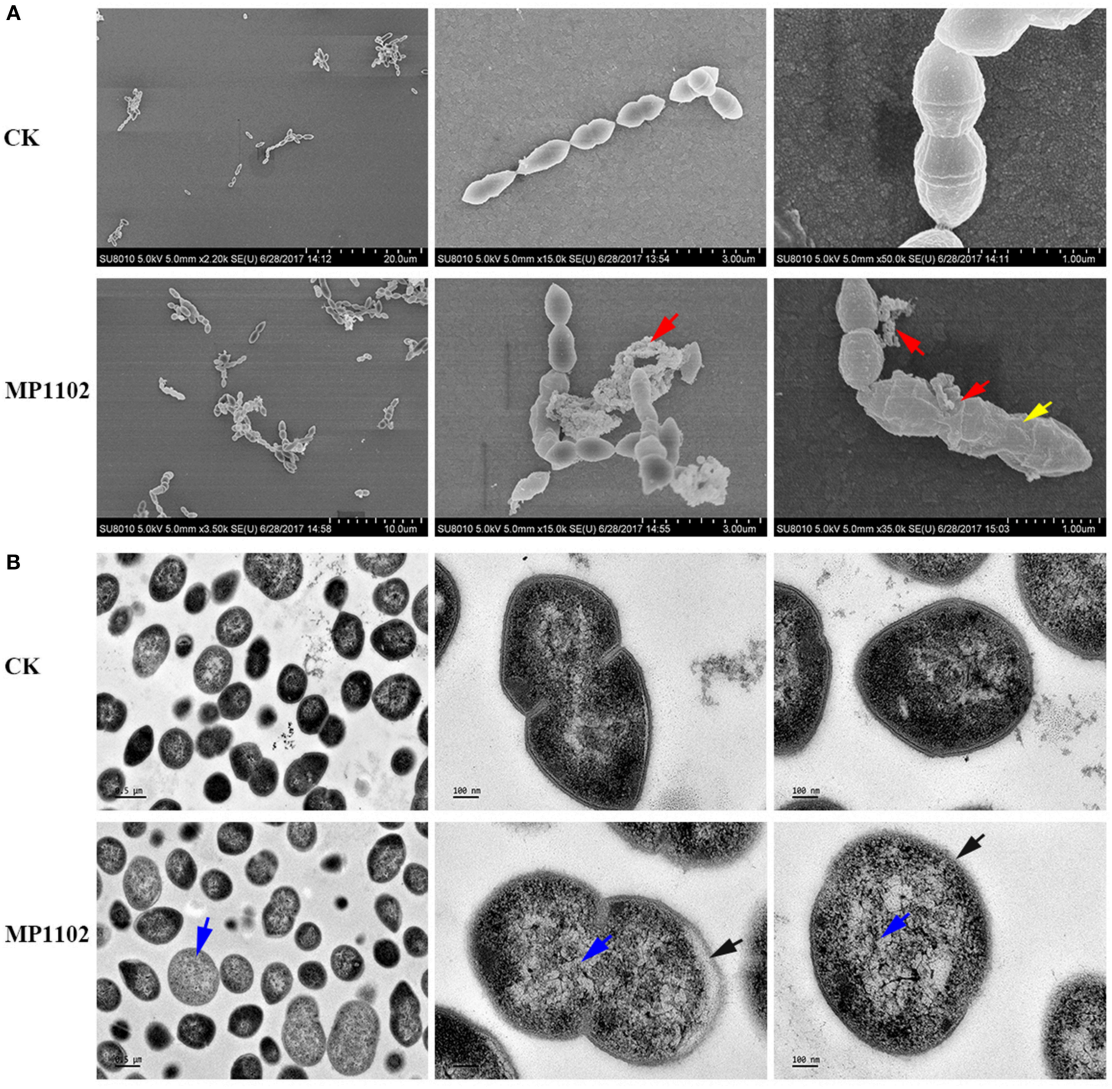
Effects of MP1102 on the cell morphology and ultrastructure of *S. suis* strain CVCC 3928. Bacteria in mid-logarithmic growth were treated with peptides at 4 × MIC for 2 h. **(A)** SEM observations. **(B)** TEM observations. Red arrows: intracellular content leakage and cell lysis; Yellow arrows: deep wrinkles on cell surface; Blue arrows: heterogeneous electron density in cytoplasm; Black arrows: incomplete plasma membrane and plasmolysis.

The effect of MP1102 on cell integrity and morphology of *S. suis* was directly visualized under SEM and TEM observations. SEM observations showed that *S. suis* cells exhibited serious cell damage after treatment with MP1102, such as deep wrinkles on the cell surface, intracellular content leakage and cell lysis (Figure 2A), compared with intact cells not treated with peptide. MP1102-treated *S. suis* cells exhibited heterogeneous electron density in the cytoplasm, an incomplete plasma membrane, and plasmolysis under TEM observations (Figure 2B). Together with the above results of bacterial plasma membrane permeabilization, these results further confirmed that MP1102 damage *S. suis* cell membranes.

### MP1102 Changed the *S. suis* Genomic DNA Conformation by DNA-Binding

**Figure 3:**
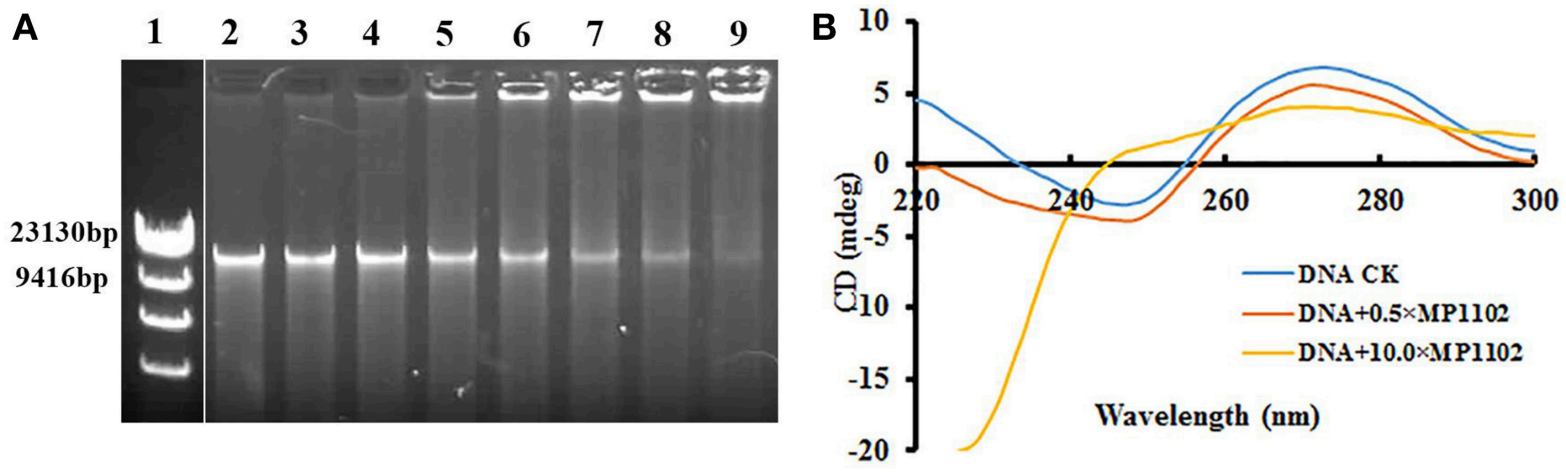
*In vitro* binding of MP1102 to the *S. suis* strain CVCC 3928 genomic DNA. **(A)** Gel retardation analysis of the binding of MP1102 to genomic DNA. Line 1: DNA maker λ DNA/*Hind*III; Lines 2–9: the mass ratios of MP1102 and genomic DNA were 0, 0.5, 1.0, 2.0, 4.0, 6.0, 8.0, and 10.0, respectively. **(B)** CD spectra analysis of genomic DNA. The mass ratios of MP1102 and genomic DNA were 0, 0.5, and 10.0, respectively.

The DNA-binding ability of MP1102 was evaluated by DNA gel retardation assay to identify potential targets in cells. As shown in Figure 3, MP1102 started to prevent the migration of genomic DNA from *S. suis* CVCC 3928 at the peptide/DNA mass ratio of 0.5. The DNA-binding activity of MP1102 was enhanced as the peptide mass increased. When the mass ratios reached up to 10.0, almost complete retardation of the genomic DNA was observed.

CD assays were performed for further confirmation of the DNA-binding affinity of MP1102. In Figure 3B, the CD spectra of *S. suis* genomic DNA showed a positive band and a negative band at ~270 and 245 nm, respectively. However, dramatic changes occurred after treatment with MP1102. The elliptic intensity of the positive band declined as the peptide content increased, suggesting the insertion of MP1102 into base pairs weakened stacking interaction. In addition, the lack of a negative band demonstrated the disappearance of the helical structure when the peptide to DNA mass ratio reached 10.

### MP1102 Protected Mice From *S. suis* Infections

#### Protection of Mice Against a Lethal Bacterial Challenge

**Figure 4:**
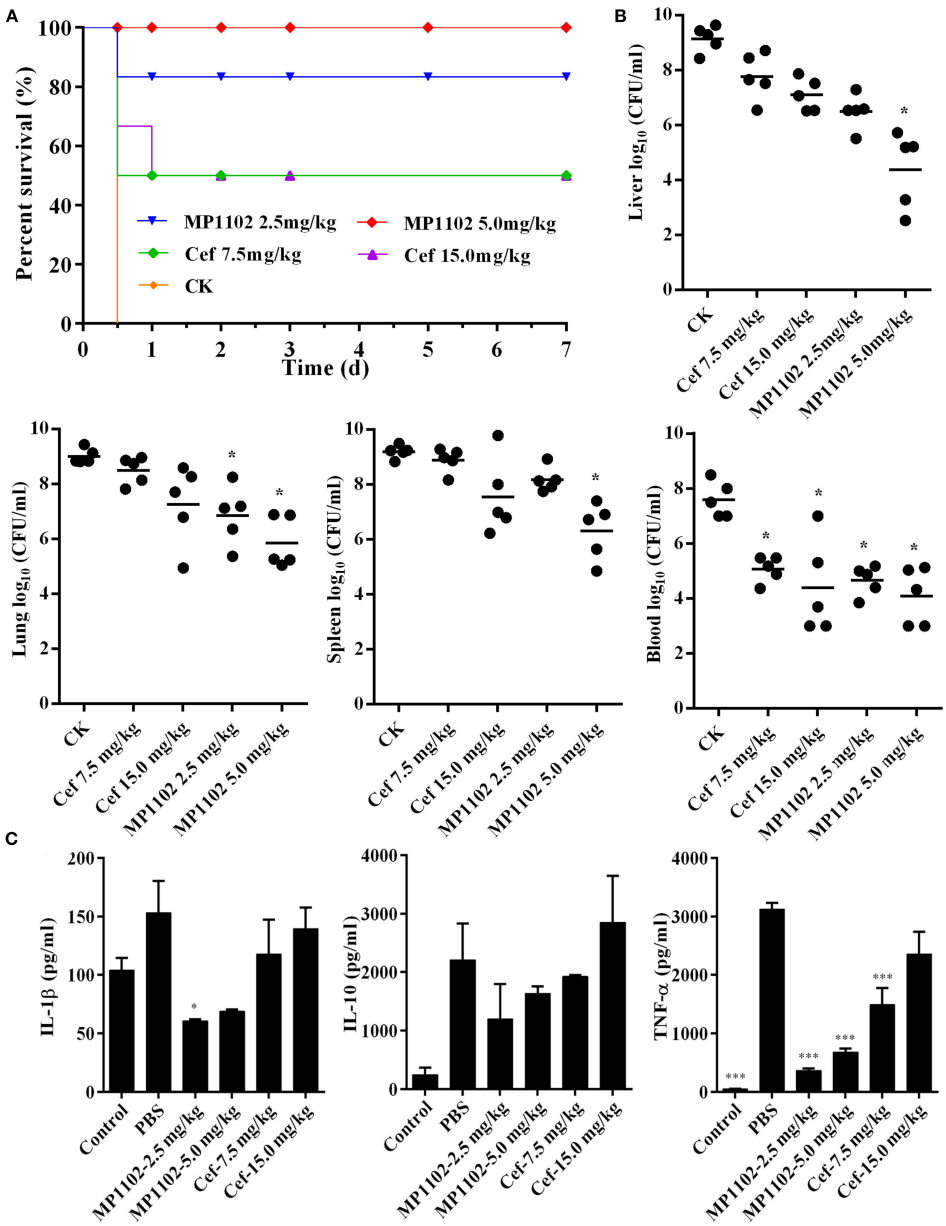
*In vivo* efficacy of MP1102 on *S. suis* infection mice. Mice were infected intraperitoneally with *S. suis* CVCC 3928 (7.5 × 10^8^ CFU/ml) and treated intraperitoneally with MP1102 and ceftriaxone at 2 h after postinfection. **(A)** Survival of mice. Survival of mice was recorded for seven days. **(B)** Effect of MP1102 on bacterial loads in liver, lung, spleen, and blood of *S. suis*-infected mice. Lungs and livers were removed at 1 d posttreatment to analyze bacterial translocation. Data are expressed as the mean ± standard deviation. *p < 0.01. **(C)** Effects of MP1102 on sera cytokines. Sera were collected and cytokines were detected at 6 h after treatment. Ceftriaxone and PBS served as positive and negative controls, respectively. ***p < 0.001.

Mice without treatment were dead within 12 h after intraperitoneal inoculation with *S. suis* CVCC 3928. In the positive control group, the survival rates of mice treated with 7.5 and 15.0 mg/kg ceftriaxone were both 50% (Figure 4A). After treatment with 2.5 and 5.0 mg/kg MP1102, the survival rates of mice were 83.3 and 100%, respectively (Figure 4A), which is higher than that of ceftriaxone. These results suggested that MP1102 could protect mice from a lethal *S. suis* challenge.

#### Inhibition of Bacterial Translocation

At the start of therapy, *S. suis* CVCC 3928 cell counts in the liver, lung, spleen, and blood were 9.14, 9.01, 9.19, and 7.60 log_10_ CFU/g, respectively (Figure 4B). After treatment with 5.0 mg/kg MP1102 for 24 h, the bacterial counts in mice liver, lung, spleen, and blood were all reduced significantly by 4.76, 3.15, 2.89, and 3.50 log_10_ CFU/g, respectively, compared with untreated mice (Figure 4B). Compared with ceftriaxone (7.5 and 15.0 mg/kg) and MP1102 (5.0 mg/kg), 5.0 mg/kg MP1102 more effectively reduced the bacterial burden; approximately 99.99% of *S. suis* cells were killed in liver, lung, spleen, and blood.

#### Inhibition of Proinflammatory Cytokines

To verify whether the protective effect of MP1102 was related to inflammatory cytokines, serum levels of IL-1β, IL-10, and TNF-α in mice infected with *S. suis* were measured. IL-1β, IL-10, and TNF-α levels in infected mice treated with 2.5 mg/kg MP1102 were 60.60, 1193.82, and 365.21 pg/ml, respectively, and those of 5.0 mg/kg MP1102 were 68.81, 1631.09, and 677.90 pg/ml, respectively. These levels were obviously lower than those from the PBS group (152.92, 2206.35, and 3118.90 pg/ml, respectively) and 15.0 mg/kg ceftriaxone group (139.43, 2848.03, and 2355.10 pg/ml, respectively). This result indicated that MP1102 inhibited the secretion of proinflammatory cytokines TNF-α and IL-1β as well as the production of anti-inflammatory cytokine IL-10 (Figure 4C).

#### Suppression of Acute Lung, Liver, and Spleen Injury

**Figure 5:**
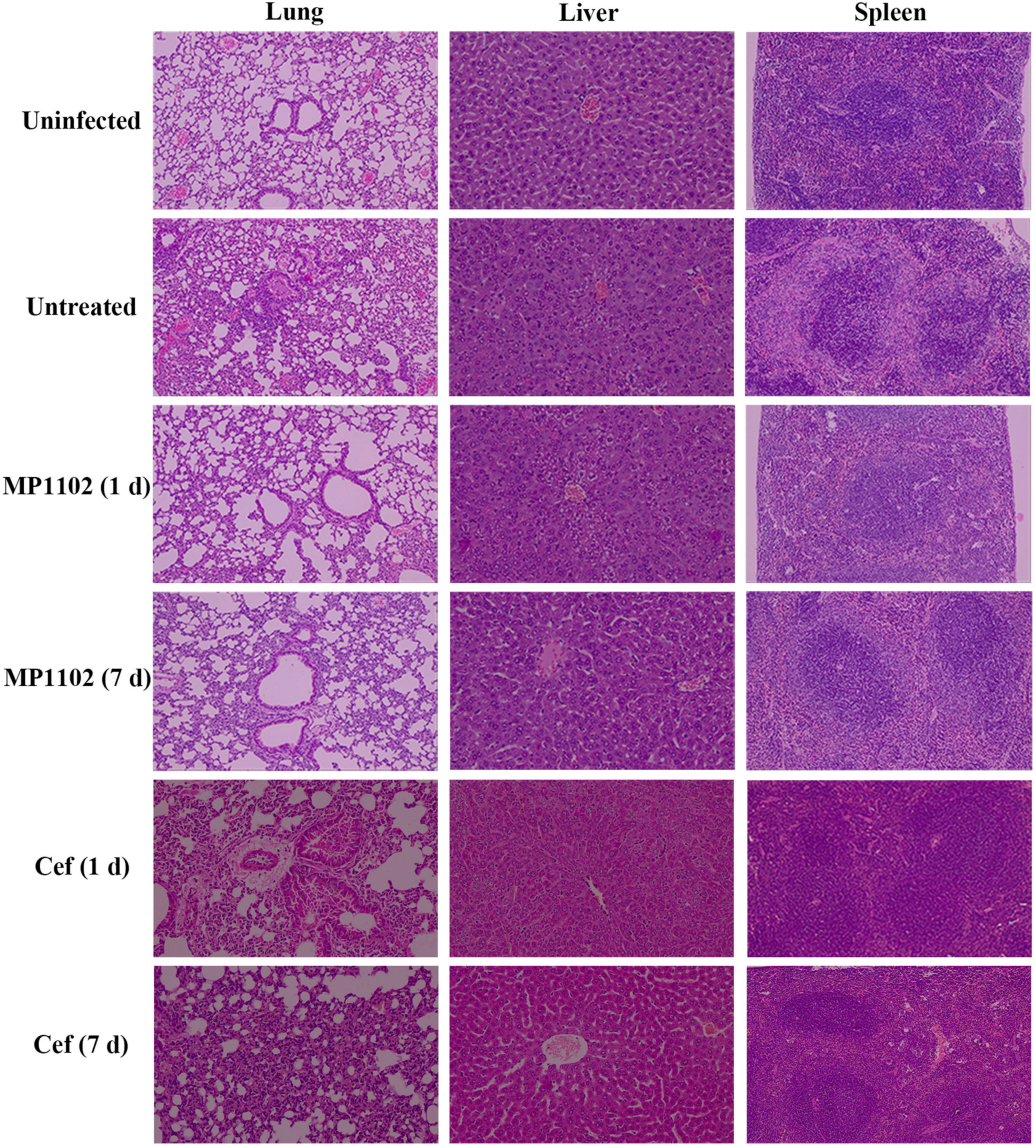
Histological analysis of effects of MP1102 and ceftriaxone on organ injury. Mice were infected intraperitoneally with *S. suis* CVCC 3928 (7.5 × 10^8^ CFU/ml) and treated with MP1102 (5.0 mg/kg) or ceftriaxone (15.0 mg/kg) at 2 h post infection. Lungs, livers, and spleens were harvested and detected at 1 and 7 d after treatment.

In the liver tissue, compared with the uninfected group (Figure 5), the morphology of hepatocytes in the untreated (PBS treated) group was irregular. In addition, >33% vacuolar degeneration was noted around the hepatic duct area, and inflammatory cells infiltrated in the hepatic sinusoid space (Figure 5) with an inflammatory degree of 3 (Table 3). After MP1102 treatment for 1 d, inflammation did not decrease obviously (Figure 5). After 7 d, inflammatory symptoms were significantly reduced, a small number of inflammatory cells infiltrated (Figure 5), and the inflammation degree was between 1 and 2 (very slight) (Table 3). For the ceftriaxone group, the surviving mice showed consistent therapeutic effects, and the scores of inflammation degree were minor (1) at 1 d and very slight (1–2) at 7 d.

**Table 3:** Degree of inflammation (score) of tissue sections.

**Organs**	**Blank control**	**Negative control**	**MP1102**		**Ceftriaxone sodium**	
	**1 d**	**1 d**	**1 d**	**7 d**	**1 d**	**7 d**
Liver	0	3	1	0	1	0
Lung	0	3	1	1	2	1
Spleen	0	3	1	1-2	2	1

*Mice were infected intraperitoneally with S. suis CVCC 3928 (7.5 × 10^8^ CFU/ml) and treated with MP1102 (5.0 mg/kg) and ceftriaxone (15.0 mg/kg) at 2 h post infection, respectively. Lungs, livers, and spleens were harvested and detected at 1 d and 7 d after treatment. Inflammatory degrees were evaluated from 3 to 0 grades (3, very serious; 2, serious; 1-2: very slight; 1, minor; 0, no inflammation phenomenon)*.

Compared with the uninfected group, diffuse infiltration of inflammatory cells in was noted in interstitial tissue of lung in the untreated (PBS treated) group (Figure 5). In addition, inflammation was noted in bronchi and perivascular areas, and narrowing of alveolar cavity was obvious with the inflammatory scores of 3 (very serious) (Table 2). After treatment with 5.0 mg/kg MP1102 for 1 d, the interstitial tissue of lung tissue was infiltrated by inflammatory cells with a slight widening of the interval and relatively normal alveolar structure (Figure 5). Inflammation degree scores (1) were minor. After 7 d, only a small amount of interstitial lung tissue was found scattered in the inflammatory cells, and the alveoli and the alveolar septum tended to be normal (Figure 5); Slight inflammation (degree 1–2) was noted. Ceftriaxone exhibited poor therapeutic efficacy in surviving mice. The score was serious (degree 3) at 1 d and minor (degree 1) at 7 d (Figure 5 and Table 3).

In the spleen tissue, compared with the normal spleen, the germinal center was obvious in the PBS group, and the edge band was clearly visible and obviously enlarged (Figure 5). Inflammation in the body was very serious (degree 3) (Table 3). After treatment with MP1102 for 1 d, the inflammatory reaction was not reduced (Figure 5), and the scores of inflammation degree were minor (degree 1) (Table 3). The splenic nodule was slightly enlarged, and the marginal area was unclear, indicating that the inflammation disappeared after 7 d of treatment (Figure 5), and the inflammation degree scores were very slight (degree 1–2). Surviving mice in the ceftriaxone treatment group showed a poor therapeutic effect, as demonstrated by the inflammation degree score of 2 (serious) at 1 d and 1 (minor) at 7 d (Figure 5 and Table 3).

These data and observations indicated that MP1102 could protect mice from *S. suis* infections *in vivo*.

## Discussion

Inappropriate antibiotic use is a major trigger of multidrug-resistant *S. suis* strain emergence, which increased treatment difficulty and complexity. AMPs, as novel antimicrobial compounds, have been proposed and widely accepted in medical application. Plectasin and its derived peptides have potential activity against *Staphylococcus* and *Streptococcus*, especially against some strains resistant to vancomycin and penicillin (Andes et al., 2009; Ostergaard et al., 2009; Brinch et al., 2010; Xiong et al., 2011). MP1102 showed 8-fold higher antibacterial activity against *S. suis* than that of plectasin (MIC: 0.454 μM) and similar levels to that of NZ2114 (MIC: 0.028–0.057 μM) and MP1106 (MIC: 0.03–0.06 μM) (Zhang et al., 2010; Cao et al., 2015; Jiao et al., 2015) (Table 2). The antibacterial activity of MP1102 was only tested against only three *S. suis* strains, and other clinical isolates may or may not be susceptible to MP1102. *S. pneumoniae* is an important human pathogen that causes pneumonia, meningitis, bacteremia, and acute otitis media in both children and adults worldwide (van der Poll and Opal, 2009; Lynch and Zhanel, 2010); In particularly, it is also likely represents a new potential zoonotic pathogen in companion animals and horses as newly reported (Ginders et al., 2017). In addition, antimicrobial resistance of *S. pneumoniae* has dramatically escalated over the past three decades (Lynch and Zhanel, 2010). Based on the strong antibacterial activity of MP1102 against *S. pneumoniae* (MIC = 0.228 μM, Table 2), the details of its special relationship with *S. pneumoniae* should be assessed in future work. MP1102 can completely kill *S. suis* CVCC 3928 cells within 6-8 h, and no colonies were recovered even at 1 × MIC (Figure 1A). Compared with other plectasin-derived peptides, MP1102 showed a superior bactericidal effect. For example, the regrowth of *S. suis* appeared after treatment with NZ2114 at 1 × and 2 × MIC for 3–4 h (Jiao et al., 2017), and the bacterial counts decreased 1.68 log_10_ CFU/ml even at 16 × MIC MP1106 during 6 h and then exhibited regrowth (Jiao et al., 2015). In our previous study, the toxicity of MP1102 in human erythrocytes was < 0.05% hemolytic activity at 128 μg/ml (Zhang et al., 2015). Given its high bactericidal efficiency and low toxicity, MP1102 preliminarily demonstrated potential use in internal medicine.

A report from Thailand showed that a *S. suis* serotype 2 strain isolated from the peritoneal fluid of a *S. suis* peritonitis patient was resistant to most antibiotics, including penicillin, but was susceptible to vancomycin (Vilaichone et al., 2000). It appears enigmatic that microbes rarely develop high-level AMP-resistance mechanisms similar to those that render many therapeutic antibiotics inefficient after short periods of application (Sahl and Shai, 2015). In our study, repeated treatment of *S. suis* CVCC 3928 with sub-MIC MP1102 did not induce the development of drug resistance. However, the MIC for ceftriaxone and penicillin increased by 2-fold, and resistance to lincomycin exhibited a 4-fold increase over the 30 d duration (Figure 1B). Remarkably, ceftriaxone, penicillin, and lincomycin are common antibiotics that have been used for many years in clinical; therefore, whether the characteristic of not easy to produce resistance of MP1102 can stand the test of time needs further studies.

PAE, as an important pharmacodynamic parameter, should be considered in the selection of antibiotic dosing regimens in clinical use. In this study, the PAE of 1 ×, 2 ×, and 4 × MIC MP1102 to *S. suis* CVCC 3928 were 2.714, 3.125, and 2.071 times increased compared with ceftriaxone (Figure 1C). A long PAE of MP1102 might contribute to lower dose, longer interval of administration, and thus potentially lower treatment cost, drug exposure, and drug resistance (Osterberg and Blaschke, 2005).

Combination therapies have distinct advantages over monotherapies in terms of their broad spectrum and synergistic effect, which directly leads to reduced dosage and indirectly leads to the delay of antibiotic resistance. Upadhyay et al. reported that a combination of ampicillin and azithromycin improved effectiveness in a murine Group B *Streptococcus* (GBS) sepsis model (Upadhyay et al., 2017). Our findings showed that MP1102 displayed synergistic or additive effects with lincomycin, penicillin, and ceftriaxone (FICI = 0.29–0.96) (Figure 1D), which suggests that the use of MP1102 with these drugs might prevent or delay the growth of resistant mutant strains.

Plectasin-derived peptide MP1102 and NZ2114 and the hybrid peptide LHP7 based on plectasin can destroy the cell membrane of Gram-positive bacteria, such as *Clostridium perfringens* and *S. aureus* Xi et al., 2014; Zong et al., 2016; Zheng et al., 2017. The *in vitro* mode action of MP1102 on the *S. suis* cell membrane was first identified in this study. The PI-stained percentages of *S. suis* CVCC 3928 cells were 34.9–58.2% after treatment with MP1102 at 1 ×, 2 ×, and 4 × MIC for 2 h, which suggested that MP1102 could induce *S. suis* cell plasma membrane damage (Figure 1E). Further analysis demonstrated that cell ultrastructure changes were accompanied by deep cell surface shrinkage, intracellular content leakage, heterogeneous electron density in cytoplasm, plasmolysis, the loss of membrane integrity and even complete cell lysis based on SEM and TEM observations (Figure 2). Combined with FACS analysis of MP1102-treated bacteria, the results revealed that the *S. suis* cell membrane was an important target of MP1102.

It has been demonstrated that AMPs have a role in binding with intracellular macromolecules after traversing the cell membrane barrier and translocating into the cytoplasm, causing inhibition of their biological synthesis and functions (Li et al., 2013; Hao et al., 2017). Genomic DNA from *S. suis* CVCC 3928 was almost completely inhibited by MP1102 at the peptide/DNA mass ratio of 10.0 on the gel (Figure 3A). The result indicated that MP1102 could tightly bind to DNA and block the migration of DNA, which is consistent with that of the peptides, such as melittin, marine arenicin, and bovine lactoferricin (Wei et al., 2016; Hao et al., 2017; Wang et al., 2017). Moreover, CD spectroscopy analyses further confirmed that MP1102 not only binds to *S. suis* CVCC 3928 genomic DNA but also interacts with *S. suis* genomic DNA, weakening base stacking force and disrupting the DNA helical structure by inserting the base pairs of DNA (Figure 3B). It has been demonstrated that some AMPs can inhibit cellular functions by binding to nucleic acids. For instance, the binding of the antimicrobial lysine-peptoid hybrid LP5 led to inhibition of DNA synthesis and induced an SOS response in *S. aureus* (Gottschalk et al., 2013). The direct interaction of host defense peptide-mimic oligo-acyl-lysyls (OAKs) with bacterial DNA inhibited the process of thymidine incorporation, thus inhibiting biosynthesis and ultimately leading to cell death (Sarig et al., 2011). The *in vitro* mechanism of MP1102 against *S. suis* is spatiotemporal. First, cell membrane is slightly destroyed by mechanical or chemical mechanisms. Then, the peptide enters into the cell and binds to genomic DNA of *S. suis*, which affects DNA replication and interferes with the cell cycle mainly by a biological process (Zong et al., 2016; Zheng et al., 2017). Finally, severe damage to the membrane occurs with the leakage of cell contents, and cell lysis is an irreversibly comprehensive result. This dual mechanism that destroyed cell membrane and interfered with DNA in terms of time and space laid the foundation for low molecular cytology and no drug resistance to MP1102.

In addition, MP1102 demonstrated excellent *in vivo* activity against *S. suis* in mice in this study (Figures 4, 5) similar to NZ2114 (Jiao et al., 2017). A mass of *S. suis* CVCC 3928 cells was detected in the liver, lung, spleen, and blood of the untreated group, which caused septicemia and acute death (Lun et al., 2007). A 33.3-50% increase in survival was noted for MP1102 treatment compared with ceftriaxone (Figure 4A), and MP1102 protected the lung, liver, and spleen from acute injury (Figure 5). A greater reduction of viable bacteria in liver, lung, spleen, and blood was also observed with increasing doses of MP1102 from 2.5 to 5.0 mg/kg of body weight compared with control mice, which showed a dose-dependent effect (Figure 4B). Moreover, MP1102 significantly inhibited the release of proinflammatory cytokines IL-1β and TNF-α in mice challenged with *S. suis* (Figure 4C), but its inhibitory effect on immunosuppressive factor IL-10 was not significant. These findings indicated that MP1102 mainly acted on the immune enhancing factor, thereby reducing the body's inflammatory response to infection. This result is similar to the report of Jiao et al. (2017), in which NZ2114 partly inhibited or regulated the serum concentrations of pro-inflammatory (IL-6 and TNF-α) and anti-inflammatory cytokines (IL-10). Together, these results suggest that MP1102 has potential to treat *S. suis* infection.

**Figure 6:**
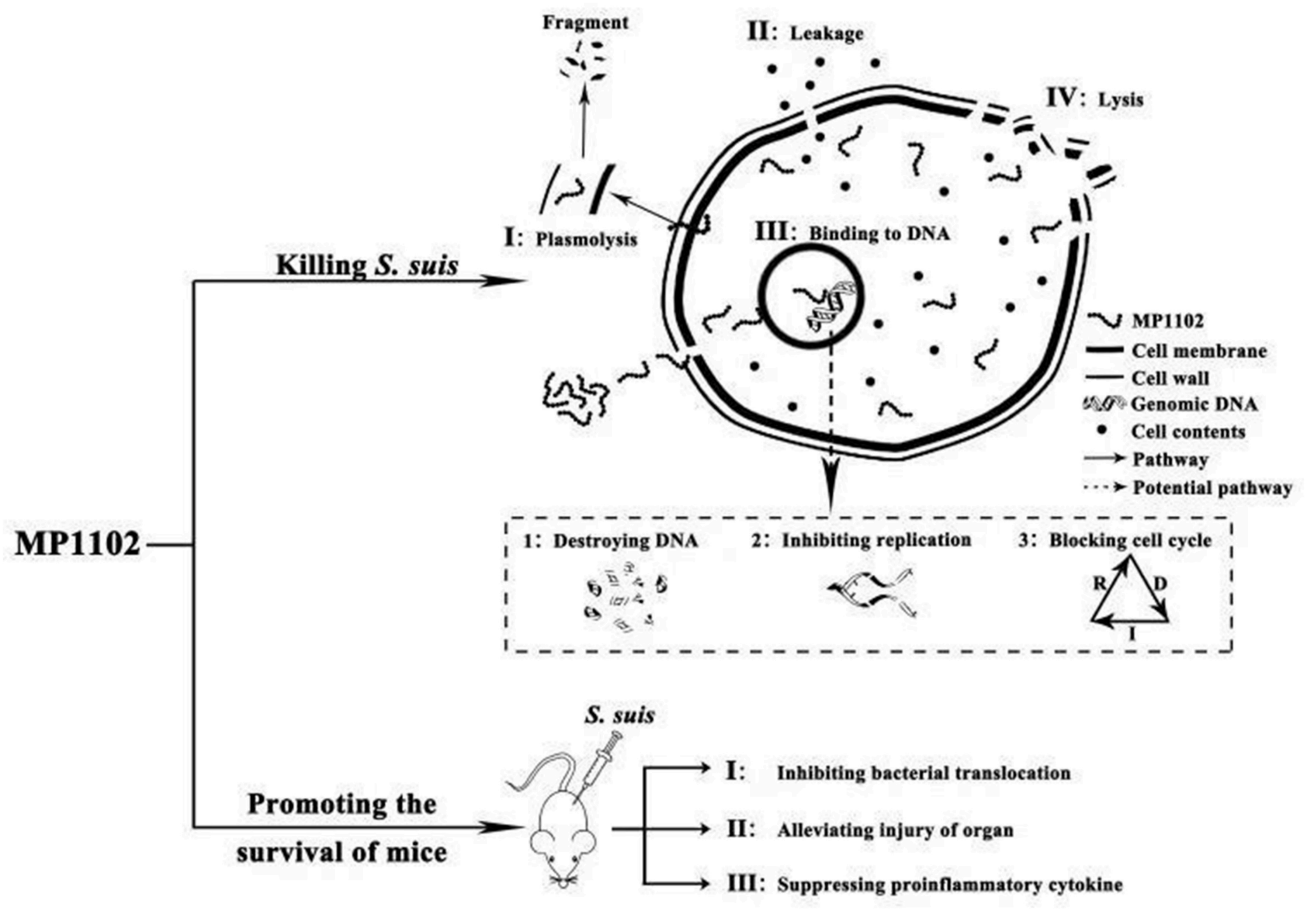
Mode of actions of MP1102 against *S. suis in vitro* and its action effects on mice challenged with *S. suis*. *In vitro* mechanism of MP1102 against *S. suis* is spatiotemporal. First, the cell membrane of the pathogen was slightly destroyed by MP1102, and the latter penetrated cell membrane and bound to genomic DNA of *S. suis*, resulting in DNA destruction, inhibition of DNA replication and cell cycle interference. As a result, severe damage of the membrane occurred, causing leakage of cell contents and cell lysis as an irreversibly comprehensive cyto-event. At the same time, *in vivo* mechanism of MP1102-protected mice challenged with *S. suis* could be accordingly deduced as inhibition of bacterial translocation, suppression of proinflammatory cytokines and alleviation of organ injury. These events ultimately contributed to improvement of mouse survival.

In conclusion, multiple *in vitro* and *in vivo* effects of MP1102 on resistant *S. suis* CVCC 3928 were studied for the first time. The following results were observed: (I) multiple modes of action of MP1102, including damage to the bacterial cell plasma membrane and interaction with bacterial genomic DNA, were revealed (Figure 6). (II) MP1102 increased the survival ratio of *S. suis*-infected mice; inhibited bacterial translocation in liver, lung, spleen, and blood; protected the lung, liver, and spleen from acute injury; and suppressed the secretion of pro-inflammatory cytokines (IL-1β and TNF-α) (Figure 6). Therefore, as a novel antimicrobial agent, MP1102 is a promising candidate against multidrug-resistant *S. suis*.

## Author Contributions

JW, DT, and HF conceptualized the study and designed the experiments. FZ and NY oversaw the operation of the experiments. FZ, NY, XMW, XW, RM, YH, and ZL conducted the data analysis and created the methodology. FZ and ZL contributed to the visualization. FZ wrote the original draft of the manuscript. JW, DT, and XMW contributed to the writing, review, and editing of the manuscript.

### Conflict of Interest Statement

The authors declare that the research was conducted in the absence of any commercial or financial relationships that could be construed as a potential conflict of interest.
